# Case Report: Nasal acinic cell carcinoma in a cat: clinicopathological and immunohistochemical characterization of a rare neoplasm

**DOI:** 10.3389/fvets.2026.1812312

**Published:** 2026-05-14

**Authors:** María Victoria Soto-López, Miguel Fuertes, Miguel Fernández, María Carmen Ferreras

**Affiliations:** 1 Departamento de Medicina, Cirugía y Anatomía Veterinaria, Facultad de Veterinaria, Hospital Veterinario-Universidad de León, León, Spain; 2 Departamento de Sanidad Animal, Facultad de Veterinaria, Universidad de León, León, Spain; 3 Instituto de Ganadería de Montaña, CSIC-Universidad de León, León, Spain

**Keywords:** acinic cell carcinoma, cat, clinical case, immunohistochemistry, nasal neoplasia, pathology

## Abstract

Acinic cell carcinoma (ACC) is a malignant epithelial neoplasm characterized by serous acinar differentiation and is most described in the salivary glands of humans and domestic animals. In animals, ACC is rare and its occurrence in the nasal cavity of cats is exceptionally uncommon. This case describes the clinical presentation, gross pathological findings, histological features and immunohistochemical profile of a nasal acinic cell carcinoma in a 14-year-old domestic shorthair cat. The animal showed chronic unilateral nasal discharge and epiphora. Bacteriological culture of nasal secretions yielded *Pasteurella* spp. and despite antimicrobial therapy the clinical condition worsened. Post-mortem examination revealed a whitish mass destroying the nasal turbinates extending to the frontal sinus. Histologically, the tumor exhibited solid, microcystic and follicular growth patterns, with moderate cellular atypia and cytoplasmic PAS-positive granules. Immunohistochemical analysis demonstrated diffuse positivity for pan-cytokeratin, with differential expressions of cytokeratin 8 and S-100 protein depending on the growth pattern, while *α*-smooth muscle actin was negative in neoplastic cells. These findings are consistent with biphasic acinic cell carcinoma showing mixed acinar and ductal differentiation. There are scant histological and immunohistochemistry complete descriptions of nasal acinic cell carcinoma in the feline species. This case states the importance of considering this rare entity in the differential diagnosis of chronic unilateral nasal disease, particularly, in older cats.

## 1 Introduction

Nasal and paranasal sinus tumors are relatively uncommon in cats, representing approximately 1–8.4% of all feline neoplasms and they predominantly affect geriatric animals, with a mean age at diagnosis exceeding 10 years (1). More than 90% of feline nasal tumors are malignant, with lymphoma being the most frequently reported histological type, followed by carcinomas, particularly adenocarcinomas and squamous cell carcinomas (1, 2). Due to the nonspecific and insidious nature of clinical signs, including nasal discharge, sneezing and epiphora, diagnosis is often delayed and advanced local disease is common at presentation.

Acinic cell carcinoma (ACC) is a malignant epithelial neoplasm characterized by serous acinar differentiation and is most described in the salivary glands. In human medicine, ACC accounts for a small proportion of salivary gland tumors and most frequently arises in the parotid gland and minor salivary glands, particularly the palatine glands (3, 4). In small animals, ACC is rare and has been sporadically reported in the salivary glands of dogs, cats and pygmy hedgedhog as well as in isolated cases affecting unusual anatomical locations (5, 6).

Histologically, ACC is known for its morphological heterogeneity, exhibiting solid, microcystic, follicular and papillary-cystic growth patterns, often within the same tumor (4, 7). Immunohistochemical studies have demonstrated variable expressions of cytokeratins and S-100 protein, reflecting different degrees of ductal and acinar differentiation (3, 4, 7, 8). Tumors showing mixed histological and immunophenotypic features are often referred to as biphasic or mixed ductulus-acinar carcinomas and have been suggested to exhibit more aggressive biological behavior (7). Nasal localization of ACC in cats is exceptionally rare, with only a single case reported to date lacking immunopathological description (8).

The present work describes a rare case of nasal acinic cell carcinoma in a domestic cat, emphasizing the clinicopathological, histological and immunohistochemical characteristics of this neoplasm and discussing its differential diagnosis and biological significance.

## 2 Case description

### 2.1 Clinical presentation

A 14-year-old female domestic shorthair cat weighing 2.15 kg was referred to the Veterinary Teaching Hospital of the University of León for evaluation of chronic unilateral nasal discharge from the right nostril accompanied by persistent epiphora. According to the owner, the clinical signs had been present for several months and had progressively worsened. No significant response to symptomatic or supportive treatment had been observed prior to referral.

On clinical examination, the cat appeared lethargic, dehydrated and showed reduced appetite. Rhinoscopic examination revealed ulcerated areas affecting the nasal mucosa. Bacterial culture of the nasal secretion yielded *Pasteurella* spp.; however, antimicrobial susceptibility testing was not available. Despite antimicrobial and supportive therapy, the clinical condition of the animal continued to deteriorate, with progressive anorexia and worsening dehydration. Given the poor clinical response and unfavorable prognosis, euthanasia was elected by the owners and for humane reasons.

### 2.2 Gross findings

**Figure 1:**
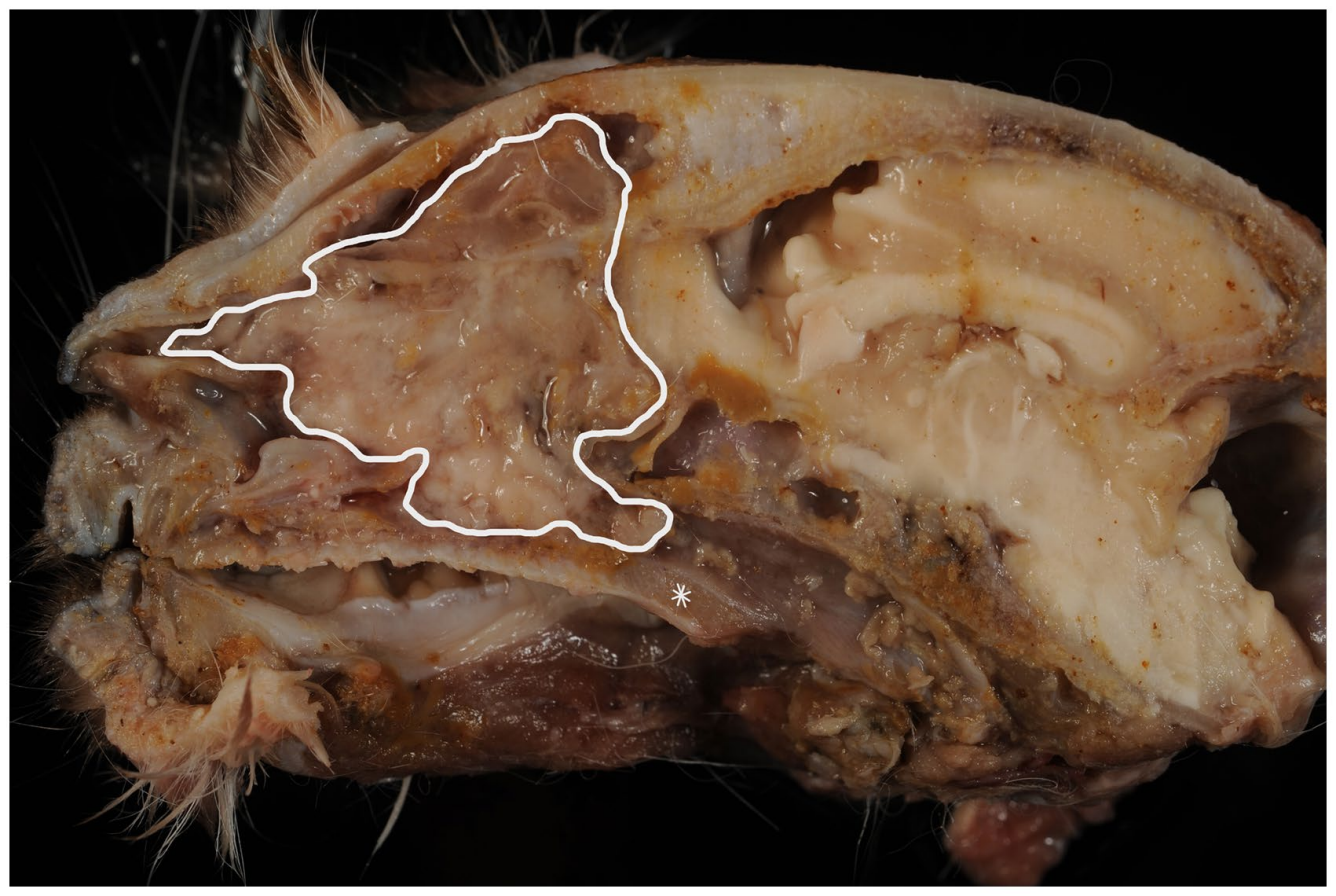
Sagittal section of the head showing the main nasal tumour mass (white line), with destruction of nasal structures. The anatomical location of the palatine salivary glands is indicated (asterisk).

A complete necropsy was performed following euthanasia. On sagittal sectioning of the head, a whitish, soft to moderately firm mass was identified within the right nasal cavity (Figure 1). The mass extensively destroyed the normal architecture of the nasal turbinates and extended into the frontal sinus. The lesion appeared locally invasive but well demarcated from adjacent tissues (Figure 1). Metastatic spread to the cranial bones or submandibular lymph nodes were not macroscopically observed.

### 2.3 Histopathology

**Figure 2:**
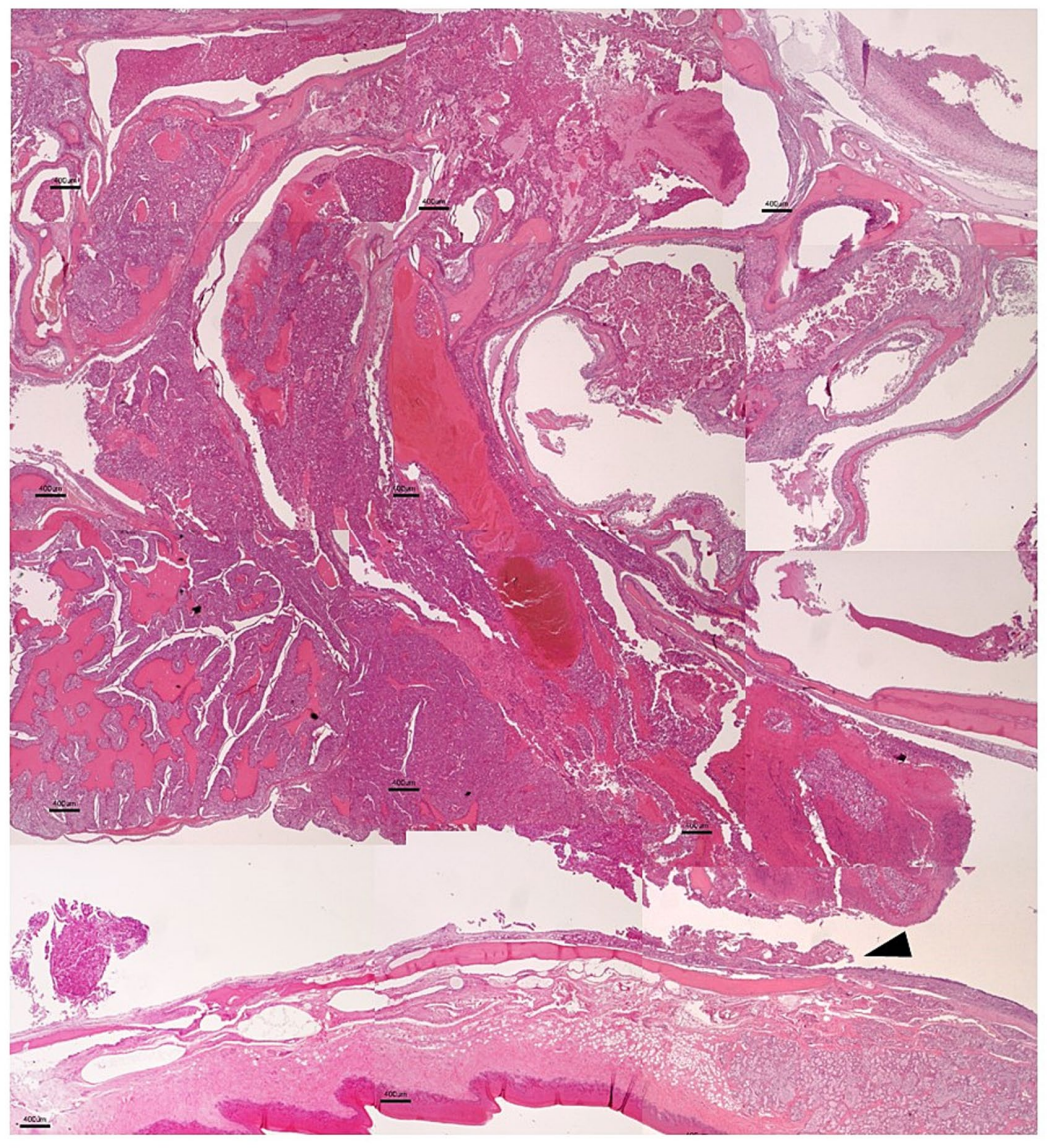
Composite histological image of the nasal tumor illustrating its anatomical relationship with the hard palate and adjacent palatine salivary glands (arrowhead). The tumor shows infiltrative growth extending toward the underlying structures. Bar = 400 μm.

**Figure 3:**
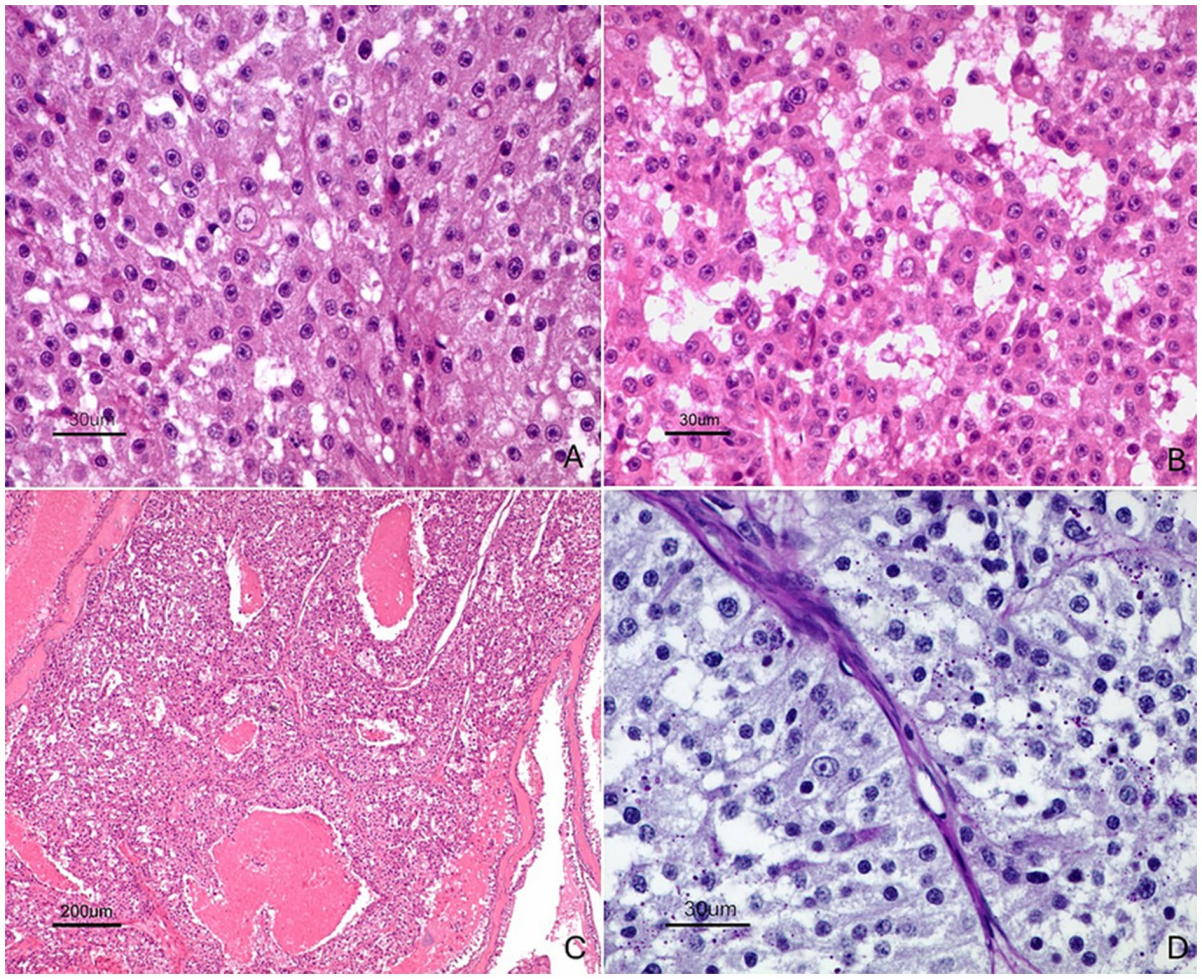
Histological features of the tumor showing different growth patterns and cytological characteristics. **(A)** Solid growth pattern composed of sheets and nests of polygonal epithelial cells with moderately abundant, lightly basophilic cytoplasm and eccentrically located round to oval nuclei, supported by scant stroma. Bar = 30 μm. **(B)** Microcystic growth pattern characterized by multiple small cyst-like spaces surrounded by neoplastic epithelial cells. Bar = 30 μm. **(C)** Follicular growth pattern consisting of irregular luminal structures lined by tumor cells and containing amphophilic material. Bar = 200 μm. **(D)** Periodic acid–Schiff (PAS)-positive intracytoplasmic granules within neoplastic cells, supporting acinar differentiation. PAS Bar = 30 μm.

Samples were routinely processed for histology. After decalcification, tissue samples from the nasal cavity and adjacent structures were processed and stained with hematoxylin–eosin and periodic acid–Schiff (PAS). Histological examination revealed an unencapsulated, infiltrative epithelial neoplasm extending from the nasal cavity to the nasopharynx (Figure 2). The tumor was composed of neoplastic cells arranged in three predominant growth patterns: solid, microcystic and follicular (Figure 3). The solid pattern consisted of sheets and nests of polygonal cells with moderately abundant, lightly basophilic cytoplasm and eccentrically located round to oval nuclei, with scant supporting stroma (Figure 3A). The microcystic pattern was characterized by small cyst-like spaces surrounded by neoplastic cells (Figure 3B), while the follicular pattern consisted of irregular cavities lined by tumor cells and filled with amphophilic material (Figure 3C). Moderate anisokaryosis was present, along with occasional atypical mitotic figures.

On routine H&E sections, the combination of an infiltrative epithelial neoplasm showing solid, microcystic and follicular architecture, together with cells containing moderately abundant granular cytoplasm, was highly suggestive of acinic cell carcinoma.

### 2.4 Special stain and immunohistochemistry

PAS staining revealed abundant intracytoplasmic PAS-positive granules within neoplastic cells, consistent with serous acinar differentiation in the solid pattern (Figure 3D). In addition to the neoplastic lesion, severe chronic rhinitis with abundant bacterial colonies was observed in adjacent nasal mucosa sampled.

The identification of PAS-positive cytoplasmic granules further supported serous acinar differentiation, a key feature favoring ACC over conventional nasal adenocarcinoma or other poorly differentiated epithelial neoplasms. Immunohistochemistry was performed to confirm epithelial lineage, further characterize the line of differentiation and assess relevant differential diagnoses.

The antibody panel, specified in Table 1, included pan-cytokeratin, selected to confirm epithelial origin; CK8 commonly expressed in ductal/luminal epithelial cells and may help identify ductal differentiation in salivary-type tumours; S-100 reported in acinic cell carcinoma and may support acinar/intercalated duct-type differentiation, and *α*-SMA to assess myoepithelial differentiation and thereby help exclude neoplasms with a myoepithelial component, such as epithelial–myoepithelial carcinoma.

**Table 1:** Expression of the markers used in the different tumour grow patterns according to the percentage of staining observed (0 = none; 1 = <25%; 2 = between 25 and 50%; 3 = 50–75%; 4 = more than 75%).

Antibody marker	Clone/Brand	D	Tumour pattern component			
			Solid	Microcystic	Follicular	Description of staining
PCK	PCK26/ Progen	1:200	1	3	1	Diffuse cytoplasmic staining in neoplastic cells
CK8	C-43/ Novus b.	1:100	0	4	4	Intense cytoplasmic staining in microcystic and follicular areas
S-100	Polyclonal/ Dako	1:1000	0–1	3	3	Positive in microcystic/follicular areas; weak or focal in solid areas
α-SMA 1A4	M0851/ Dako	1:100	0	0	0	Restricted to vascular smooth muscle/controls; negative in neoplastic cells
PAS (*)	–	–	4	0	0	PAS-positive intracytoplasmic granulation

* = histochemistry staining; D = dilution.

Formalin-fixed paraffin-embedded 3.5-μm-thick serial tissue sections was used to characterize the cells Epitope retrievals were accomplished in Tris-based solution (PT-Link System, Agilent Technologies) at pH = 9 for alpha SMA and CK8, and enzymatic with trypsin for PCK and S-100. In addition, slides were incubated overnight at 4 °C with primary antibodies diluted (PCK 1:200; CK8 1:100; S-100 1:1000 and *α*-SMA 1:100) in a phosphate-buffered saline in a humidified chamber. The next day, after washing, samples were incubated with a secondary antibody for 40 min and with 3,3-diaminobenzidine (DAB; Agilent Technologies) for 5 min. Finally, slides were rinsed in tap water and counterstained with Mayer’s haematoxylin for 10 s. Negative and positive controls, for the primary antibodies, were also included: canine smooth muscle tissue for α-SMA and normal palatine and sublingual salivary glands from the same animal served as positive controls for the immunohistochemical procedures (Supplementary Figures 1, 2). Negative controls were obtained by omission of the primary antibody. The results of IHC study in each of the neoplastic cell subtypes are summarized in Table 1.

**Figure 4:**
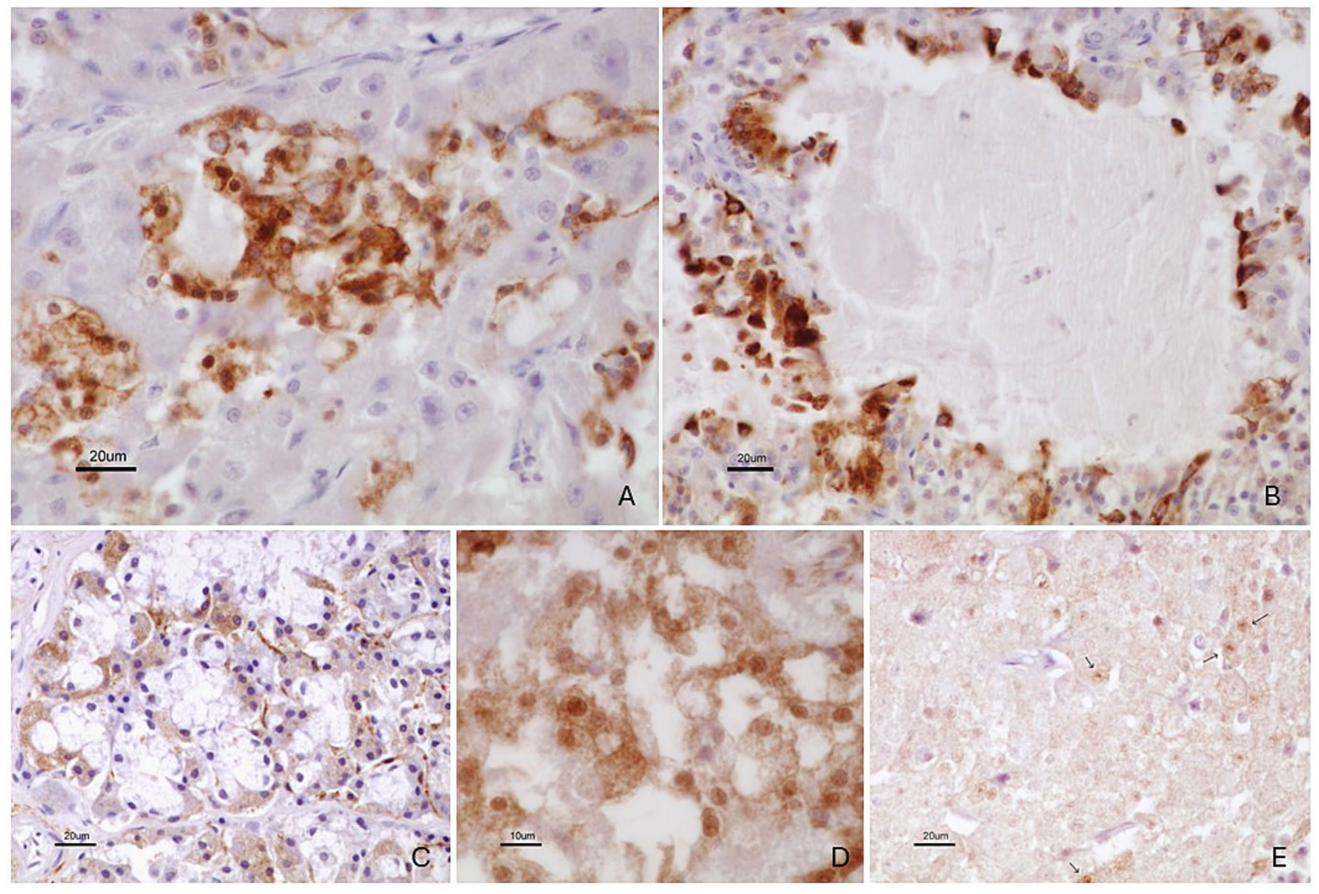
Immunohistochemical features of the tumor. **(A,B)** Cytoplasmic CK8 immunopositivity in neoplastic cells, more prominent in microcystic **(A)** and follicular **(B)** areas, highlighting ductal/luminal differentiation. Bar = 20 μm. **(C)** Strong S-100 immunopositivity in normal serous salivary gland cells (internal control). **(D)** Weak and focal S-100 immunoreactivity in tumor cells within the solid growth pattern, occasionally with nuclear staining. **(E)** More intense S-100 cytoplasmic immunopositivity in neoplastic cells within the microcystic pattern. Bars = 20 μm **(A–C)** and 10 μm **(D,E)**.

Neoplastic cells showed diffuse cytoplasmic positivity for pan-cytokeratin, confirming their epithelial origin (Supplementary Figure 3). Differential immunoreactivity was observed among the different growth patterns (Table 1). Neoplastic cells in the microcystic (Figure 4A) and follicular areas (Figure 4B) were strongly positive for CK8 and S-100 protein (Figures 4C,D, respectively), whereas cells in the solid pattern were negative for CK8 and showed only weak and occasional S-100 positivity (Figure 4E). *α*-SMA immunostaining was restricted to the smooth muscle cells of blood vessel walls within the tumor stroma (Supplementary Figure 4A), while neoplastic cells were consistently negative (Supplementary Figure 4B).

### 2.5 Diagnostic

Based on routine histology, the main differential diagnoses considered included primary nasal adenocarcinoma, other salivary gland-type carcinomas, and less likely neuroectodermal or neuroendocrine neoplasms. The presence of PAS-positive intracytoplasmic granules favored acinic differentiation. Immunohistochemistry then confirmed epithelial origin (PCK), supported ductal/acinar heterogeneity (CK8 and S-100), and excluded a significant myoepithelial component (α-SMA negativity), supporting the final diagnosis of biphasic acinic cell carcinoma.

## 3 Discussion

Nasal and paranasal sinus tumors in cats are relatively uncommon but predominantly malignant, typically affecting geriatric animals (1, 2). The clinical presentation is often insidious and nonspecific, with unilateral nasal discharge, sneezing and epiphora being among the most frequently reported signs (1). In the present case, chronic unilateral nasal discharge and persistent epiphora were the main clinical manifestations, consistent with previous descriptions of feline nasal neoplasia (8). These clinical signs are generally attributed to progressive obstruction and destruction of nasal structures, as well as compression or occlusion of the nasolacrimal duct. Importantly, such signs may closely mimic chronic inflammatory or infectious rhinitis, potentially delaying advanced diagnostic investigations and definitive diagnosis (1, 2). Acinic cell carcinoma (ACC) is an uncommon malignant epithelial neoplasm in veterinary species and its occurrence in the nasal cavity of cats represents an exceptional finding. To date, only one other case of nasal ACC has been documented in a cat, which similarly presented with chronic nasal discharge and was diagnosed through imaging and histopathology, underscoring the rarity of this tumor in the sinonasal tract of felines and avoiding immunohistochemistry characterization (8). The present case therefore provides valuable additional clinicopathological data and contributes to expanding the limited veterinary literature on this neoplasm.

Gross examination revealed a whitish, soft, locally invasive mass that destroyed the nasal turbinates and extended into the frontal sinus. Similar macroscopic features have been described in the previously reported feline nasal ACC (8) and in ACCs arising from salivary glands in both humans and animals (6, 7). Although no regional or distant metastases were detected in this case, ACCs are recognized for their locally aggressive behavior, characterized by infiltrative growth and extensive tissue destruction even in the absence of metastatic spread (7). Therefore, the biological behavior of ACC should not be underestimated based solely on the lack of detectable metastases.

Histologically, ACC is characterized by marked morphological heterogeneity, which is considered one of its defining features (4, 7). In the present case, the tumor exhibited solid, microcystic and follicular growth patterns, with a predominance of the solid and microcystic components. This morphological diversity aligns with the classic patterns including solid, microcystic, follicular and papillary-cystic —and highlights the complexity of these tumors due to their variable components (9). Recognition of this characteristic is essential to avoid misclassification, particularly in tumors arising in unusual anatomical locations such as the nasal cavity.

The identification of abundant PAS-positive intracytoplasmic granules within neoplastic cells is important, as these granules correspond to zymogen-like secretory material typical of serous acinar differentiation (7). This feature is especially useful in differentiating ACC from other primary nasal carcinomas, such as adenocarcinomas and poorly differentiated epithelial tumors, which may show overlapping architectural features but lack serous acinar characteristics (2).

Immunohistochemical findings further supported the diagnosis of ACC. Diffuse positivity for PCK confirmed the epithelial origin of the tumor, while differential expression of cytokeratin 8 (CK8) and S-100 protein was observed among the various histological patterns. Previous immunohistochemical studies of ACC arising in minor salivary glands have demonstrated a variable expression of cytokeratins, particularly CK8, between acinar and ductal components, supporting the use of this marker to distinguish tumor subpopulations and differentiation states (3, 4). In the present case, CK8 and S-100 positivity was mainly confined to the microcystic and follicular components, whereas the solid pattern was largely negative for CK8 and showed only weak S-100 expression. This immunophenotypic distribution supports classification of the tumor as biphasic, with mixed ductulus-acinar differentiation.

Biphasic or mixed ductulus-acinar ACCs, characterized by the coexistence of cytokeratin-positive ductal structures and cytokeratin-negative solid areas, have been suggested to exhibit more aggressive biological behavior compared with monomorphic tumors (7). Although prognostic data for ACC in cats are extremely limited, the extensive local invasion observed in the present case is consistent with this proposed behavior. While long-term follow-up was not available due to euthanasia, the degree of local tissue destruction suggests that early diagnosis would be critical for any potential therapeutic intervention.

The absence of *α*- SMA expression in neoplastic cells is also diagnostically relevant. In salivary gland tumors, α-SMA expression is typically associated with myoepithelial differentiation. The lack of α-SMA immunoreactivity in this case indicates the absence of a myoepithelial component, consistent with previous studies of ACC and useful in differentiating this neoplasm from other salivary gland-type tumors with myoepithelial participation, such as epithelial–myoepithelial carcinoma (4).

ACC is believed to arise from reserve cells of the intercalated ducts of salivary glands (8). In human medicine, excepting salivary glands, particularly the palatine glands, are considered a frequent site of origin for ACC (3, 9). In the present case, the close anatomical relationship between the tumor and the palatine salivary glands supports the hypothesis that the neoplasm originated from minor salivary gland tissue associated with the nasal cavity rather than from the respiratory epithelium itself.

From a differential diagnostic, nasal tumors in cats most commonly comprise lymphomas or epithelial carcinomas (1, 2). Although rarer neoplasms, such as olfactory neuroblastoma have also been described in the feline nasal cavity (10, 11), their histomorphological and immunophenotypic profiles differ markedly from those observed in ACC, reinforcing the distinct nature of the present case (12, 13).

In addition to this case, *Pasteurella* spp. was isolated from nasal secretions and severe purulent rhinitis with bacterial colonies was observed adjacent to the tumour. Chronic inflammation is reported in association with feline nasal tumors and may contribute to clinical signs and secondary complications (1). However, a direct causal relationship between *Pasteurella* spp. infection and development of acinic cell carcinoma cannot be established from a single case. In cats, chronic inflammation has been implicated in oncogenesis in selected settings, most notably feline injection-site sarcoma (14–16), supporting the concept that persistent inflammatory stimuli may contribute to neoplastic transformation in some contexts. Nevertheless, evidence specifically linking *Pasteurella* spp. to nasal epithelial or salivary-type carcinogenesis in cats is currently lacking. Although a causal relationship between chronic inflammation and tumor development cannot be established, the coexistence of both processes may further complicate the initial clinical interpretation by reinforcing an assumption of primary inflammatory or infectious disease at first.

## 4 Conclusion

In conclusion, this case highlights the importance of including rare salivary gland-type tumors, such as acinic cell carcinoma, in the differential diagnosis of chronic unilateral nasal disease in older cats. Accurate diagnosis relies on careful histopathological examination supported by targeted immunohistochemistry, particularly in unusual anatomical locations. Given the rarity of this neoplasm in cats, additional case reports are needed to better characterize its biological behavior, optimal diagnostic approach and potential therapeutic strategies.

## Data Availability

The original contributions presented in the study are included in the article/Supplementary material, further inquiries can be directed to the corresponding author.

## Supplementary material

Supplementary Figure 1 Normal salivary gland (control). Diffuse cytoplasmic pancytokeratin (PCK) immunopositivity in epithelial cells. ×300.

Supplementary Figure 2 Normal salivary gland (control). CK8 immunopositivity in serous demilunes (arrowhead) and intercalated ducts (arrow), confirming expected staining pattern. Bar = 20 μm.

Supplementary Figure 3 Tumour. Microcystic pattern shows diffuse cytoplasmic pancytokeratin (PCK) immunopositivity in neoplastic epithelial cells. ×300.

Supplementary Figure 4 α-SMA immunohistochemistry. (A) Normal salivary gland showing cytoplasmic immunopositivity in myoepithelial cells (internal control). (B) Absence of α-SMA expression in neoplastic cells, supporting lack of myoepithelial differentiation. Bars = 30 μm **(A)** and 50 μm **(B)**.

## References

[1] Mukaratirwa S van der Linde-Sipman JS Gruys E. Feline nasal and paranasal sinus tumours: clinicopathological study, histomorphological description and diagnostic immunohistochemistry. J Feline Med Surg. (2001) 3:235–45. doi: 10.1053/jfms.2001.0141, 11795961 PMC10822294

[2] Wilson DW Dungworth DL. "Tumors of the respiratory tract". In: Meuten DJ, editor. Tumors in Domestic Animals, 4th Edn. Ames, IW: Iowa State Press (2002). p. 365–80.

[3] Crivelini MM de Sousa SOM Araújo VC. Immunohistochemical study of acinic cell carcinoma of minor salivary gland. Oral Oncol. (1997) 33:204–8. doi: 10.1016/S0964-1955(96)00064-4, 9307730

[4] Li X Shi Z Wang Y Liu Y Liu T. Immunohistochemical expression of cytokeratins in human salivary gland acinic cell carcinomas. Oral Surg Oral Med Oral Pathol Oral Radiol. (2015) 120:248–57. doi: 10.1016/j.oooo.2015.04.014, 26166029

[5] Fukuzawa R Fukuzawa K Abe H Nagai T Kameyama K. Acinic cell carcinoma in an African pygmy hedgehog (*Atelerix albiventris*). Vet Clin Pathol. (2004) 33:39–42. doi: 10.1111/j.1939-165X.2004.tb00348.x, 15048626

[6] Hammer A Getzy D Ogilvie G Upton M Klausner J Kisseberth WC. Salivary gland neoplasia in the dog and cat: survival times and prognostic factors. J Am Anim Hosp Assoc. (2001) 37:478–82. doi: 10.5326/15473317-37-5-478, 11563448

[7] Schwarz S Zenk J Müller M Ettl T Wünsch H. The many faces of acinic cell carcinomas of the salivary glands: histological and immunohistological subtypes related to clinical parameters and prognosis. Histopathology. (2012) 61:395–408. doi: 10.1111/j.1365-2559.2012.04233.x, 22551398

[8] Psalla D Geigy C Konar M Café Marçal V Oevermann A. Nasal acinic cell carcinoma in a cat. Vet Pathol. (2008) 45:365–8. doi: 10.1354/vp.45-3-365, 18487495

[9] Vander Poorten V Triantafyllou A Thompson LDR Bishop J Hauben E Hunt J . Salivary acinic cell carcinoma: reappraisal and update. Eur Arch Otorrinolaringol. (2016) 273:3511–31. doi: 10.1007/s00405-015-3855-7, 26685679

[10] Wilson DW Dungworth DL. “Tumors of the Respiratory Tract,” In Veterian Key. (2020). Available online at: https://veteriankey.com/tumors-of-the-respiratory-tract/

[11] de Oliveira EC de Castro LT Paz MC Bandinelli MB Pavarini SP. Olfactory neuroblastoma in a cat. Braz J Vet Pathol. (2024) 17:213–6. doi: 10.24070/bjvp.1983-0246.v17i3p213-216

[12] Ogilvie GK LaRue SM. Canine and feline nasal and paranasal sinus tumors. Vet Clin North Am Small Anim Pract. (1992) 22:1133–44. doi: 10.1016/s0195-5616(92)50305-9, 1523785

[13] WHO Classification of Tumours Editorial Board. Head and Neck Tumours. WHO Classification of Tumours. 5th ed. Lyon: International Agency for Research on Cancer (2024).

[14] Porcellato I Menchetti L Brachelente C Sforna M Reginato A Lepri E . Feline injection-site sarcoma. Vet Pathol. (2017) 54:204–11. doi: 10.1177/0300985816677148, 28005492

[15] Hartmann K Day MJ Thiry E Lloret A Frymus T Addie D . Feline injection-site sarcoma: ABCD guidelines on prevention and management. J Feline Med Surg. (2015) 17:606–13. doi: 10.1177/1098612X15588451, 26101312 PMC11148925

[16] Jelínek F. Postinflammatory sarcoma in cats. Exp Toxicol Pathol. (2003) 55:167–72. doi: 10.1078/0940-2993-00307, 14620538

