# Supplementary figures and images for: Case Report: Nasal acinic cell carcinoma in a cat: clinicopathological and immunohistochemical characterization of a rare neoplasm
Source: Front Vet Sci. 2026 May 14;13:1812312. doi: 10.3389/fvets.2026.1812312 (PMC13218214; doi:10.3389/fvets.2026.1812312)

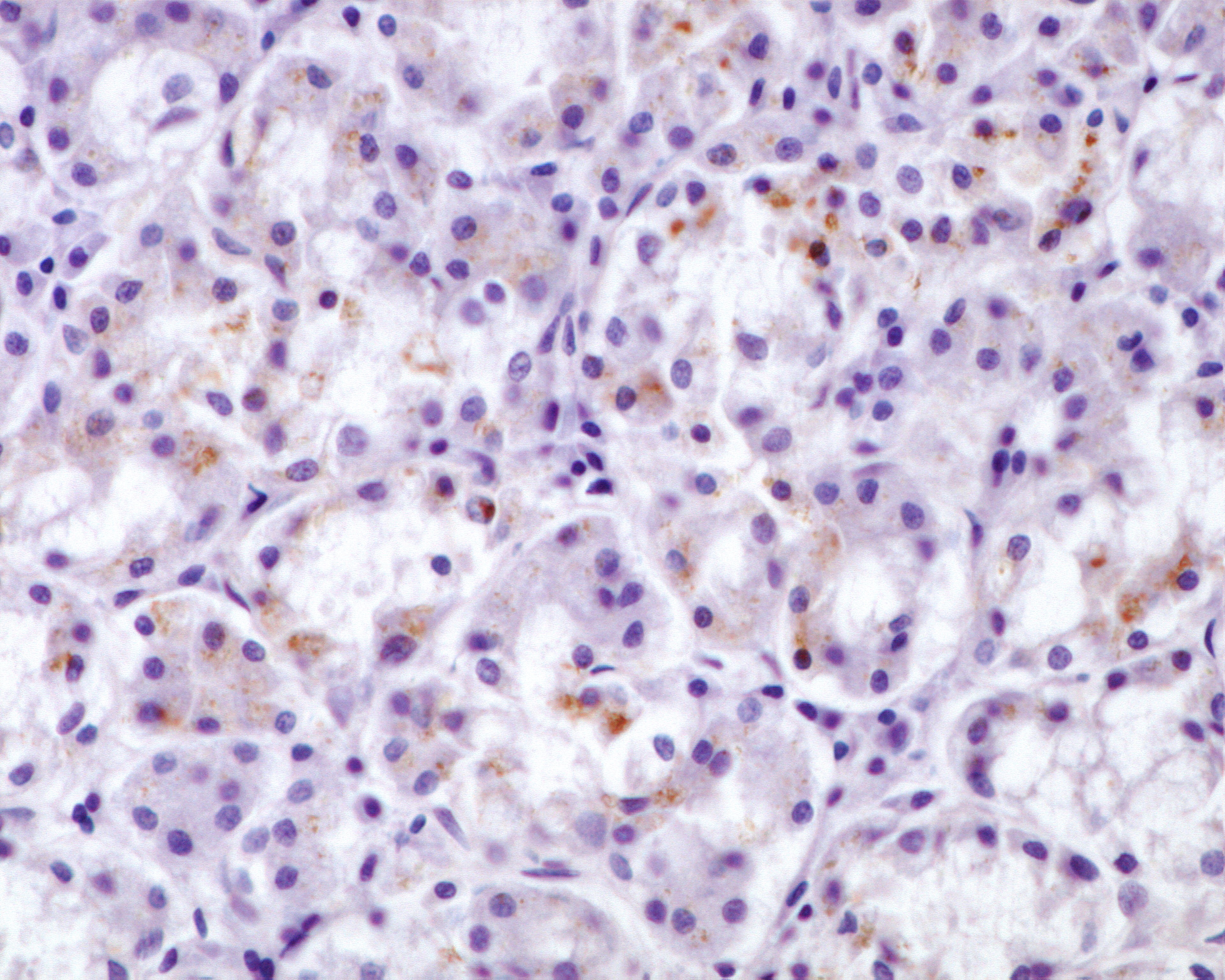

Supplement: Supplementary Figure 1 — Normal salivary gland (control). Diffuse cytoplasmic pancytokeratin (PCK) immunopositivity in epithelial cells. ×300. [file Image_1.jpeg]

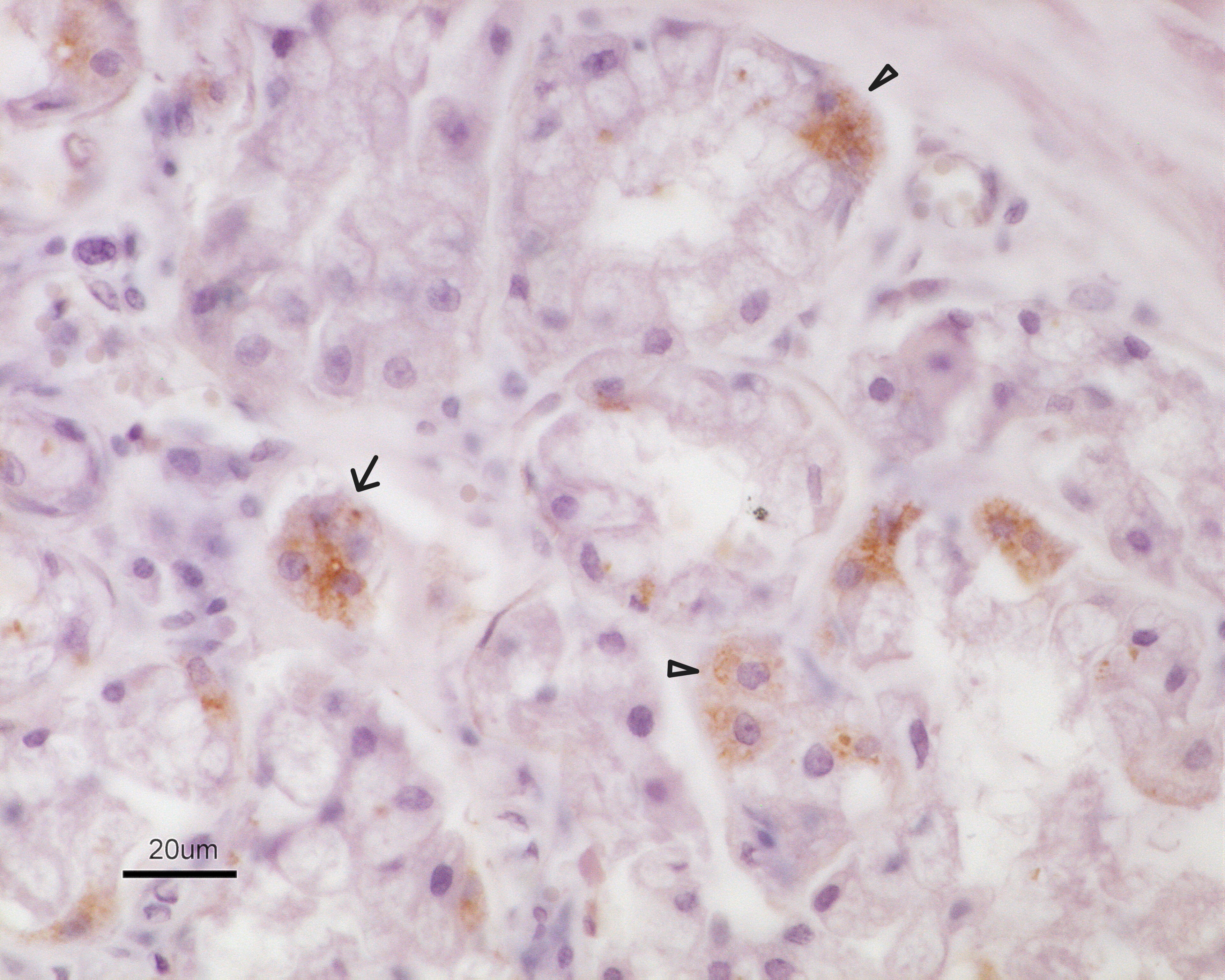

Supplement: Supplementary Figure 2 — Normal salivary gland (control). CK8 immunopositivity in serous demilunes (arrowhead) and intercalated ducts (arrow), confirming expected staining pattern. Bar = 20 μm. [file Image_2.jpeg]

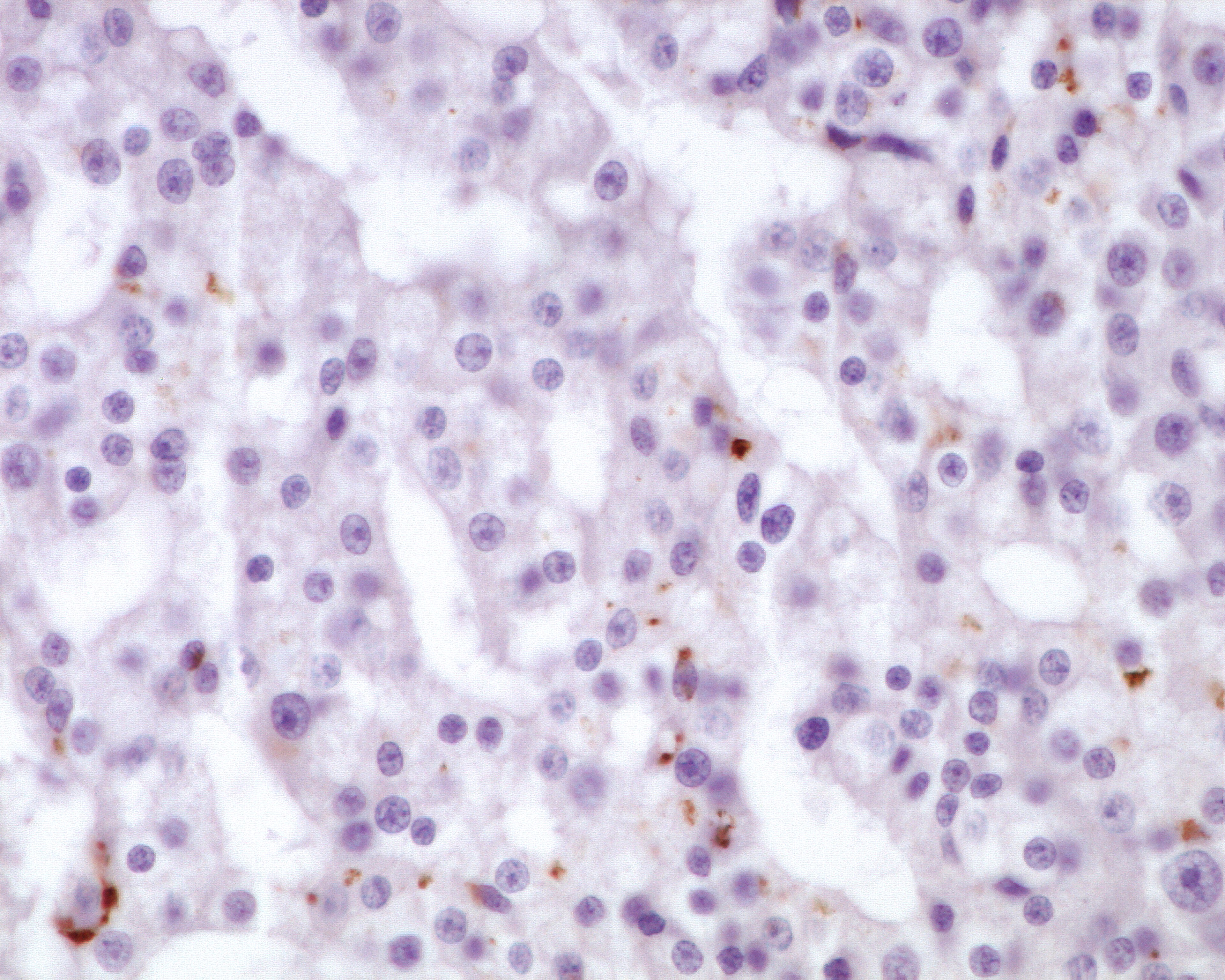

Supplement: Supplementary Figure 3 — Tumour. Microcystic pattern shows diffuse cytoplasmic pancytokeratin (PCK) immunopositivity in neoplastic epithelial cells. ×300. [file Image_3.jpeg]

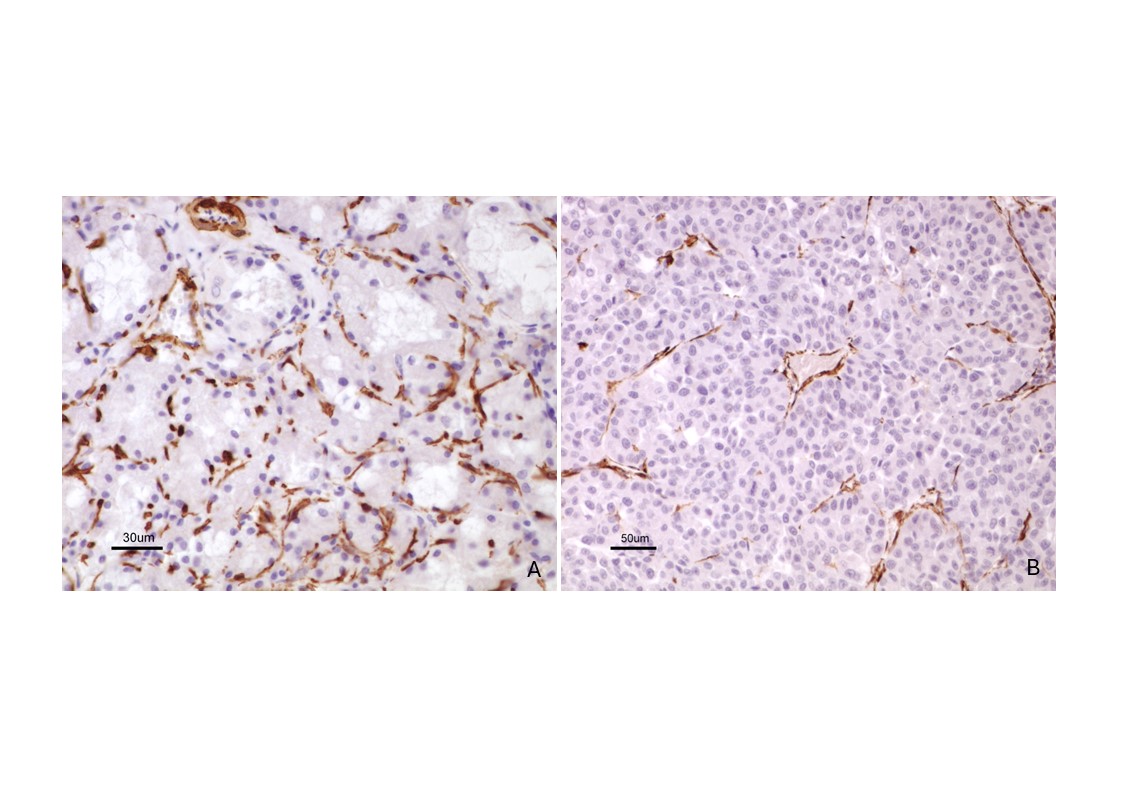

Supplement: Supplementary Figure 4 — α-SMA immunohistochemistry. (A) Normal salivary gland showing cytoplasmic immunopositivity in myoepithelial cells (internal control). (B) Absence of α-SMA expression in neoplastic cells, supporting lack of myoepithelial differentiation. Bars = 30 μm (A) and 50 μm (B). [file Image_4.jpeg]
